# Lipopeptides as main ingredients for inhibition of fungal phytopathogens by *Bacillus subtilis/amyloliquefaciens*

**DOI:** 10.1111/1751-7915.12238

**Published:** 2014-12-19

**Authors:** Hélène Cawoy, Delphine Debois, Laurent Franzil, Edwin De Pauw, Philippe Thonart, Marc Ongena

**Affiliations:** 1 Walloon Center for Industrial Microbiology, Gembloux Agro-Bio Tech, University of LiegeGembloux, Belgium; 2 Mass Spectrometry Laboratory (LSM-GIGA-R), Chemistry Department, University of LiegeLiege, Belgium

## Abstract

Some isolates of the *Bacillus subtilis/amyloliquefaciens* species are known for their plant protective activity against fungal phytopathogens. It is notably due to their genetic potential to form an impressive array of antibiotics including non-ribosomal lipopeptides (LPs). In the work presented here, we wanted to gain further insights into the relative role of these LPs in the global antifungal activity of *B. subtilis/amyloliquefaciens*. To that end, a comparative study was conducted involving multiple strains that were tested against four different phytopathogens. We combined various approaches to further exemplify that secretion of those LPs is a crucial trait in direct pathogen ward off and this can actually be generalized to all members of these species. Our data illustrate that for each LP family, the fungitoxic activity varies in function of the target species and that the production of iturins and fengycins is modulated by the presence of pathogens. Our data on the relative involvement of these LPs in the biocontrol activity and modulation of their production are discussed in the context of natural conditions in the rhizosphere.

## Introduction

Biological control through the use of natural antagonistic microorganisms has emerged as a promising alternative to reduce the use of chemical pesticides in agriculture. Some beneficial bacteria (notably belonging to the *Bacillus* and closely related *Paenibacillus* genera) living in association with plant roots are of particular interest in that context (McSpadden Gardener, 2004; Cawoy *et al*., 2011; Pérez-García *et al*., 2011). Their disease protection activity relies on three main traits. The first is a high ecological fitness regarding their ability to colonize roots, which is a prerequisite to efficiently compete for space and nutrients in the rhizosphere microenvironment. The second is their strong antagonistic activity toward various plant pathogens, which is based on the secretion of highly active antimicrobials. The third is their ability to trigger an immune reaction in plant tissues leading to a systemically expressed resistance state that render the host less susceptible to subsequent infection (induced systemic resistance or ISR phenomenon) (Lugtenberg and Kamilova, 2009; Berendsen *et al*., 2012).

Direct antagonism of phytopathogens is a key biocontrol mechanism and depends on efficient antibiotic production. Some *Bacillus* species such as *B. subtilis* and *B. amyloliquefaciens* may dedicate up to 8% of their genetic equipment to the synthesis of a wide array of antimicrobial compounds among which lytic enzymes, lantibiotics and a range of non-ribosomally synthesized (lipo)peptides and polyketides (Chen *et al*., 2009; Rückert *et al*., 2011). Such antibiotic arsenal and a high rhizosphere fitness probably explain the strong biocontrol potential of bacilli both in vitro and under field conditions and its success as marketed product (Cawoy *et al*., 2011; Kirk *et al*., 2013; Larkin and Tavantzis, 2013; Shen *et al*., 2013; Yang *et al*., 2013). Among the *Bacillus* antibiome, cyclic lipopeptides (LPs) of the surfactin, iturin and fengycin families are of high interest not only because they are produced at high rates by *B. subtilis*/*amyloliquefaciens* cells under in vitro conditions in bioreactors, but also because they are the main antimicrobials that can be secreted in biologically relevant amounts under natural conditions of growth in the rhizosphere (Kinsella *et al*., 2009; Nihorimbere *et al*., 2012; Dietel *et al*., 2013; Debois *et al*., 2014).

These cyclic LPs are formed by non-ribosomal peptide synthetases (NRPS) or hybrid polyketide synthases/NRPS. Such biosynthetic systems lead to a remarkable structural heterogeneity among the cyclic LPs products, which vary from one family to another in the type, number and sequence of amino acid residues as well as in the nature of the peptide cyclization. Within each family, some differences occur in the nature, length and branching of the fatty acid chain leading to the co-production of various homologues of surfactin, iturin and fengycin by a single strain (Ongena and Jacques, 2008; Raaijmakers *et al*., 2010). Even if some strains of the *B. subtilis/amyloliquefaciens* species are among the most efficient microbial biocontrol agents, further development as microbial soil inoculants is ampered by multiple constraints including variable efficacy across environmental conditions, and plant-pathogen systems. Resolving these constraints requires a better knowledge not only on antibiotic production *in planta* under agronomical conditions, but also on the activity/specificity of these molecules in function of the target pathogen.

The involvement of LPs in the potential of *B. amyloliquefaciens*/*subtilis* to antagonize phytopathogens has been documented but, so far, most works have been case studies involving single plant growth-promoting rhizobacteria-pathogen systems (Touré *et al*., 2004; Romero *et al*., 2007; Malfanova *et al*., 2012; Yuan *et al*., 2012) and there is a lack of information concerning the relative importance of LPs among other antibiotics and enzymes potentially involved in pathogen inhibition. In this context, the present work was initiated to appreciate whether a key role of the different LP families in direct pathogen ward off can actually be generalized to all strains of the *B. subtilis*/*amyloliquefaciens* complex. To this end, a comparative study was conducted on a range of isolates that were selected based on their diverse LP signatures. Those strains were first confronted to several phytopathogens of agronomical importance and the correlation between antagonism amplitude and the amounts of LPs accumulating in the inhibition zone yielded first indications on the relative importance of each family for the inhibition of specific pathogens. These data were supported by results from more targeted approaches such as testing selected LP mutants or via imaging the spatial distribution of the whole LP pattern in the inhibition area with matrix-assisted laser desorption ionization (MALDI)-time of flight (TOF) mass spectrometry (MS). Our results also strongly suggest that the LP-based antagonistic potential of these bacilli can be impacted by unexpected phenomena such as a modulated production upon perception of the fungal pathogen or a pathogen-dependent co-precipitation of the different LPs. All these data are interpreted and discussed for their biological relevance in the contexts of rhizosphere ecology and biocontrol.

## Results and discussion

### Confrontation tests reveal specific involvement of the various LP families in fungal antagonism

**Figure 1:**
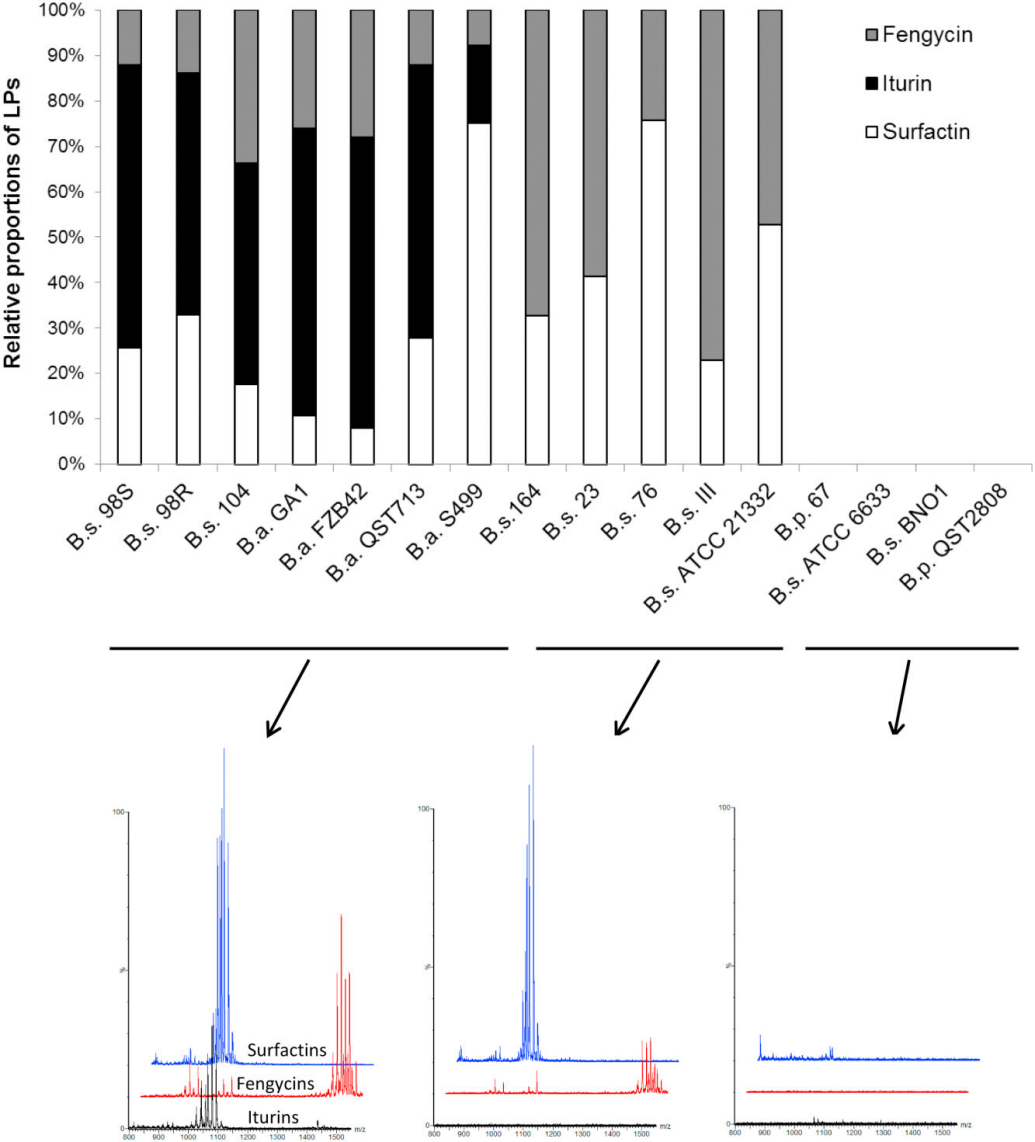
Relative proportions of the LPs surfactin, fengycin, iturin in the different supernatants. Strains were cultivated in Opt medium for 3 days. Obtained supernatants were analysed by UPLC-MS. Presented data are results of one representative experiment. A biological repetition of the assay provided similar results.

We first performed a comparative study involving a range of *Bacillus* strains isolated from the phytosphere and belonging to the species *B. subtilis (B.s.), B. amyloliquefaciens (B.a.) and B. pumilus (B.p.)*. These isolates were selected according to their different LP signatures, which were determined in agitated cultures using a rich medium optimized for production of these compounds. Some isolates did not produce any LPs; others produced two or all three families LPs, including iturins (or their bacillomycin-type variants), fengycins and surfactins, in specific relative proportions (Fig. 1). Based on these results, strains were subdivided into three groups: producers of the three families of LPs (like 98S and QST713), producers of surfactin and fengycin but not iturin (like ATCC21332 and 164) and isolates incapable of LP production (such as BNO1) (Fig. 1).

**Figure 2:**
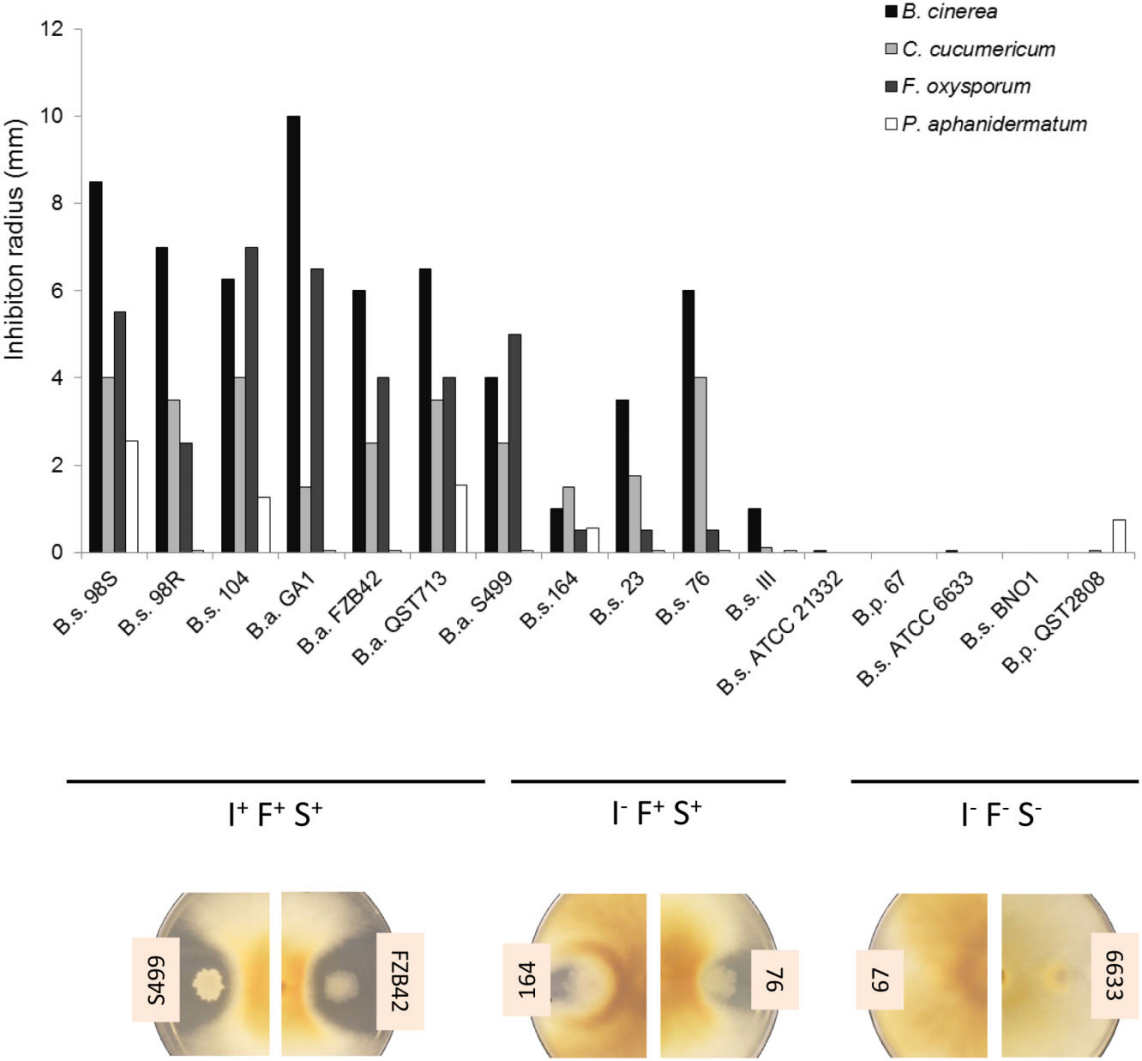
Antagonism potential of the 17 strains when confronted with pathogens. Based on LP production in the inhibition zone, the strains are divided into three groups: producers of the three families of LPs (I^+^F^+^S^+^), producers of fengycin and surfactin (I^-^ F^+^S^+^), non-LP producers (I^-^ F^-^ S^-^). Antagonism intensity was evaluated in direct confrontation tests by measuring the radius of the inhibition zone between the plant growth-promoting rhizobacteria (PGPR) and the fungi. Presented data are means from two biological repeats.

All strains were compared for their capacity to inhibit a set of agronomically important infectious fungi including two foliar pathogens, *Cladosporium cucumerinum* and *Botrytis cinerea*, and two soil-borne pathogens, *Fusarium oxysporum* and *Pythium aphanidermatum*. The antagonistic potential was evaluated based on the size of inhibition zones in direct confrontation tests on Potato Dextrose Agar (PDA) plates. A first analysis of data presented in Fig. 2 showed that strains co-producing the three LP families were globally the most efficient in pathogen inhibition (strain cluster I^+^F^+^S^+^) compared with strains that do not produce iturin (I^-^ F^+^S^+^) and those not able to form any LPs. Iturin appeared to be the main component involved based on the significant decrease in antagonistic activity between strain clusters I^+^F^+^S^+^ and I^-^ F^+^S^+^. This phenomenon is not only most markedly observed for *Fusarium* antagonism, but is also tangible for the other pathogens (Fig. 2). However, in these conditions, the LP profiles of the strains could have been modified compared with liquid cultures involving planktonic cells growing in the presence of other nutrients in the optimized medium (Fig. 1). Agar plugs were thus collected from the diffusion zone close to the colonies developing on PDA and liquid chromatography-MS analyses of LPs present in these samples revealed similar patterns for each strain compared with liquid cultures (Fig. S1).

**Figure 3:**
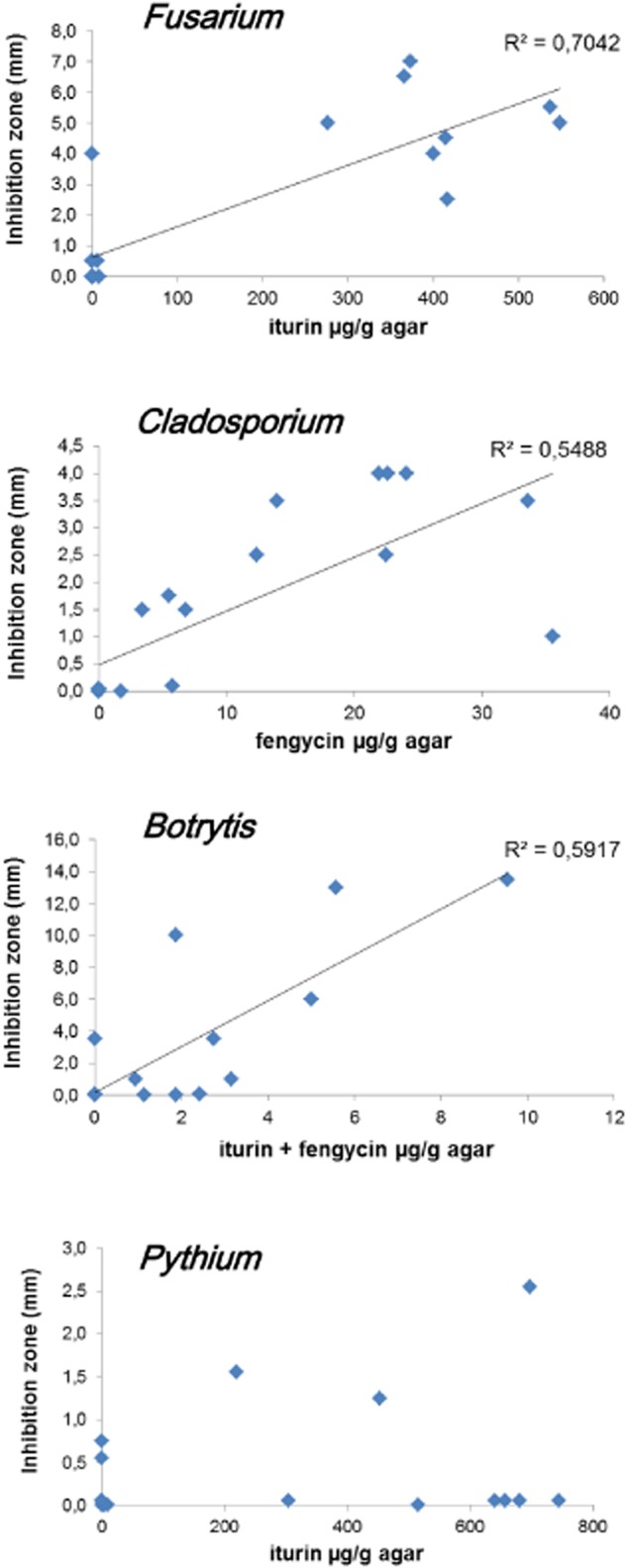
Correlations between LP concentration in the inhibition zone and intensity of the antagonism. Agar samples were taken from the inhibition zone, extracted with 50% ACN and analysed for LP content by UPLC-MS. Presented data are means from two biological repeats.

On that basis, we wanted to establish some correlation between antagonism intensity and iturin/fengycin concentrations in the inhibition zone. Figure 3 displays the combinations that led to consistent correlations (see also Fig. S2), further exemplifying that inhibition of *F. oxysporum* depended on iturin and fengycin, whereas the antagonism against *C. cucumericum* mainly relied on fengycin production. *Pythium aphanidermatum* was only slightly inhibited by a few strains, and no clear correlations could be established with LP concentrations in the medium surrounding bacterial colonies. As the cell wall of oomycetes is mainly composed of cellulose and not chitin as observed for true fungi, we tested possible involvement of bacterial cellulases in the observed inhibitory effect. However, the semi-quantitative cellulase assays revealed that most of the studied strains secrete this enzyme to a similar level that does not correlate with their differential inhibitory activities on the pathogen (Fig. S3). For *B. cinerea*, inhibition correlated well with total fengycin, and iturin amounts as far as low concentrations are considered (Fig. 3 and Fig. S2).

**Figure 4:**
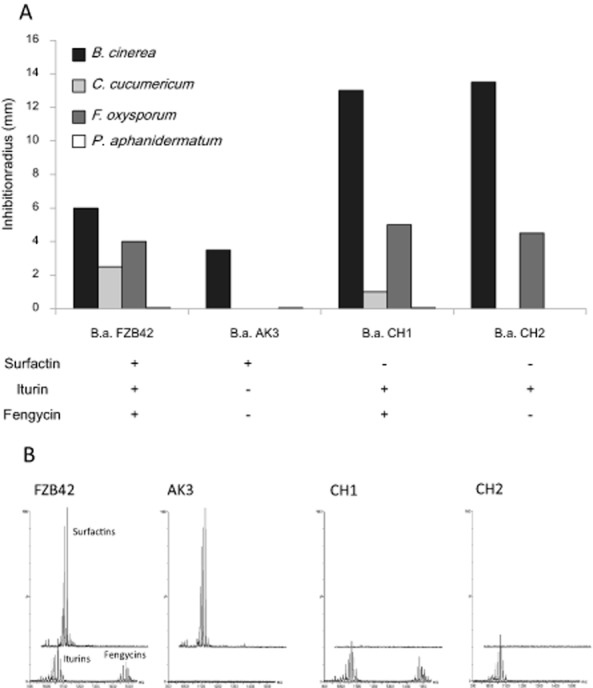
A. Intensity of the antagonism displayed by strain *B.a*. FZB42 and its lipopeptide mutants against pathogens. Presented data are means of two biological repeats. B. UPLC-MS chromatograms illustrating the differential LP production of *B.a*. FZB42 and its mutants. Shown data are representative of two biological repeats.

In order to confirm the specific involvement of LPs in the studied inhibitions, we used several mutants of strain FZB42 repressed in LP synthesis. The wild-type secretes all kinds of LPs and clearly inhibits three pathogens but not *Pythium*. As shown in Fig. 4, most of this antagonistic activity is lost in the AK3 derivative impaired in biosynthesis of bacillomycins (iturin variants) and fengycins. However, this mutant is still capable of inhibiting *Botrytis*, which is obviously not due to surfactin as previous data demonstrated that this compound is not toxic for this fungus (Malfanova *et al*., 2012). In agreement with data presented in Fig. 3, CH1 and CH2 mutants retained a similar inhibitory effect on *Fusarium* compared with the wild-type and displayed an even enhanced antagonism toward *Botrytis* (see below), supporting the crucial role played by iturin and fengycin in the inhibition of these two pathogens (Fig. 4). As expected, the CH2 derivative lost its inhibitory effect on *Cladosporium* mainly caused by fengycins. The antagonism developed by the wild-type FZB42 against *Botrytis* was related to the formation of a white line in the inhibition zone surrounding colonies of the wild-type FZB42, but this was not for mutants CH1 and CH2. Preliminary analysis of agar samples collected in the area corresponding to the line suggested that it could originate from co-precipitation of the three LPs once they reach a certain concentration, resulting in lower amounts of soluble iturin and fengycin diffusing in the medium and involved in pathogen arrest (Fig. S3). Such a phenomenon has already been observed for *Pseudomonas* LPs in the interaction of tolaasins with White Line Inducing Principle (WLIP) (Wong and Preece, 1979) as well as for sessilins and orfamides (D'aes *et al*., 2014).

Iturins and fengycins are thus important factors for fungal inhibition (Ongena and Jacques, 2008; Falardeau *et al*., 2013), and collectively, these results show that they are the major *Bacillus* metabolites involved in antagonism of *F. oxysporum, C. cucumerinum* and *B. cinerea*. There is no clear involvement of iturin or fengycin in the inhibition of the oomycete *Pythium aphanidermatum*. Surfactins are massively released by all LP-producing strains tested here but are poorly involved in pathogen antagonism. It displays some antibiotic activity (Bais *et al*., 2004; Xu *et al*., 2014), but has only rarely been identified as to have direct significant impact on fungal growth at biologically relevant concentrations (low μM range) (Angelini *et al*., 2009; Raaijmakers and Mazzola, 2012). Moreover, we further exemplify some specificity regarding the target species. As occasionally reported, iturins and fengycins may interfere with some intracellular processes in fungal plant pathogens notably leading to inhibition of toxin formation (Hu *et al*., 2009). However, the most evidenced effect of these LPs is the disruption of membrane integrity, leading to the lysis of various fungal life stages like mycelium, conidia or zoospores for oomycete pathogens (Raaijmakers *et al*., 2010). The efficacy of this cell disruption tightly depends on the lipid composition of the target membrane as well illustrated in the case of surfactin, which is poorly fungitoxic, but clearly exerts potent bactericidal and virucidal effects and is also active on plant cells, at least for some species (see below). More generally, variations in the potential of *Bacillus* LPs to disturb the integrity of biological membranes or artificial mimicking lipid (mono)bilayers has been extensively demonstrated and it has been reviewed recently by Falardeau and colleagues (2013). Such specificity regarding the target membrane could explain our observations on the variability of fungitoxicity level for each LP in function of the antagonist species. Additional research is needed to fully understand the precise mechanisms of action and membrane determinants in cell sensitivity to fengycin and iturins (including mycosubtilins and bacillomycins) but it still clearly appeared that it depends on both the anionic nature of phospholipids, the presence of sphingomyelin and the type and content in sterols (Avis and Bélanger, 2001; Deleu *et al*., 2008; Eeman *et al*., 2009; Nasir and Besson, 2011).

### Modulation of secreted LP amounts in the presence of pathogens

**Figure 5:**
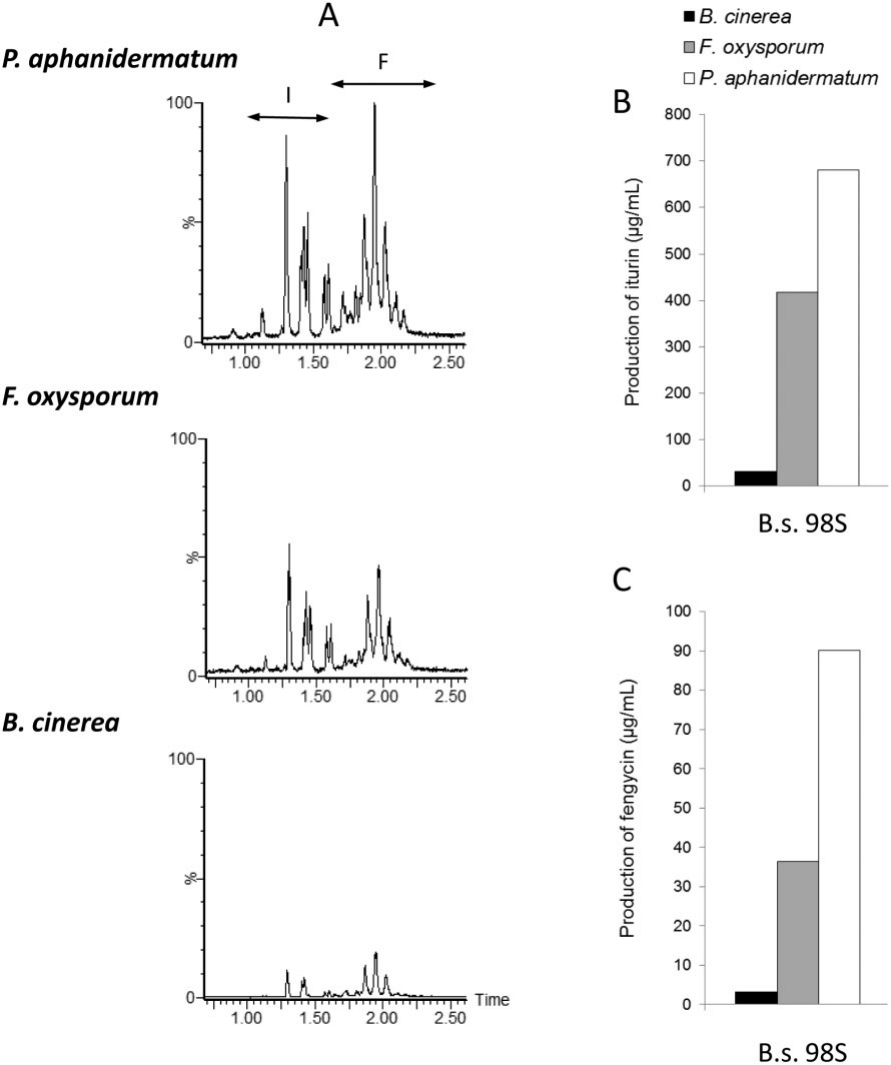
Impact of the encountered pathogen on the LP concentration in the inhibition zone.A. Chromatograms illustrating the differential LP production by strain 98S in contact with *Pythium aphanidermatum*, *Fusarium oxysporum* and *Botrytis cinerea*.B. Iturin and C. fengycin production by strain 98S in contact with different pathogens. Similar data were obtained for all studied isolates. Agar samples were taken from the inhibition zone and analysed for LPs content. Presented data are means of two biological repeats.

Interestingly, considering x scales in Fig. 3 and Fig. S2, it appeared that for most *Bacillus* strains, very different amounts of LPs accumulated in the inhibition zone depending on the nature of the pathogen encountered. This is detailed in Fig. 5 for strain 98S as representative of producers of the three families. A much higher production of iturins and fengycins was observed upon incubation in the presence of *Pythium* and *Fusarium* compared with *Botrytis* for which no increased LP accumulation could be noticed. As all these antagonism tests were conducted under the same conditions, bacterial colonies exhibited a visually similar growth when confronted to each pathogen. These observations strongly suggest that some chemical signal emitted (no contact) by the pathogens could be perceived by the bacteria which in turn modulates antibiotic synthesis. It has been occasionally reported that LP production is qualitatively and quantitatively modulated by various external factors inherent to the rhizosphere ecology such as the specific nutritional status imposed by root exudation, a reduced oxygen availability, a neutral to acidic pH or a low temperature (Nihorimbere *et al*., 2009; Pertot *et al*., 2013). However, very little is still known about cues from other soil-inhabiting microbes that may also impact the expression of these antibiotics in plant beneficials. In that context, our data suggest that *B. amyloliquefaciens* can sense some signals emitted by certain phytopathogens resulting in enhanced production of iturins and fengycins. Bacterial isolates readily interact with fungi sharing the ecosystem and antibiotic production may be modified as described with *Pseudomonas chlororaphis* PCL 1391 in which phenazine biosynthesis is repressed by fusaric acid secreted by *F. oxysporum* (van Rij *et al*., 2005). However, to our knowledge, such a modulation of antibiotic production in *Bacillus* upon fungal perception is a new concept with possibly high impact for biocontrol (Frey-Klett *et al*., 2011). These data open doors to further exploring a new type of microbial interactions and seeking for molecules with a role in cell-to-cell communication between bacilli and fungal pathogens.

### Iturins are produced on root exudates to inhibitory amounts as revealed by MS imaging

All experiments described above were performed by using synthetic media, which do not reflect the real nutritional status of bacteria evolving in the rhizosphere both qualitatively and quantitatively. In this environment, available nutrients are almost exclusively provided by root exudates with specific compositions in organic acids, sugars or amino acids. As root exudation rate is limited, rhizosphere microbes are in a nutrient-starved physiological state compared with rich culture media (Bais *et al*., 2006; Nihorimbere *et al*., 2009; 2012). We thus wanted to test the production of antimicrobial LPs under the nutritional status imposed by the plant i.e. by growing the bacterium in the presence of root exudates as sole carbon source. These exudates were collected from hydroponic cultures of plants grown for 4 weeks in the greenhouse. For these experiments, we selected strain 98S because it co-produces the three LP families (Fig. 1) and based on its high inhibitory activity on PDA (Fig. 2). 98S was therefore tested for antagonism against *F. oxysporum* on plates filled with gelified root exudates released by several plants including bean, zucchini and tomato. The bacterium was still capable of inhibiting the fungus under these conditions with 58 ± 12%, 29 ± 2% and 34 ± 5% growth reduction observed respectively on zucchini, bean and tomato exudates.

**Figure 6:**
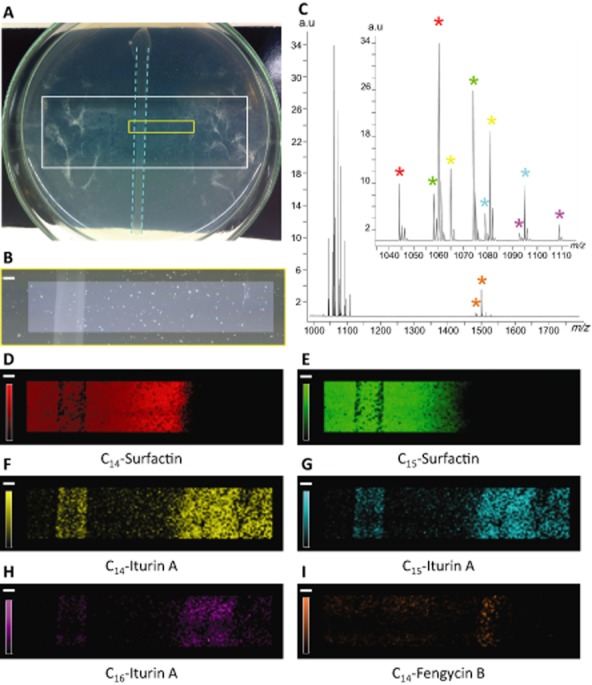
MALDI-MS imaging reveals the involvement of LPs in *B.a*. 98S antagonism against *F. oxysporum*.A. Picture of the confrontation plate, containing the Indium-Tin Oxide (ITO)-coated glass slide (white rectangle) on which strain 98S was streaked in the middle of the slide (blue dotted lines) and left to incubate during 2 days. *Fusarium* was then inoculated close to and at distance of the bacterial colony and left to incubate three more days. After drying and matrix coating, the molecular content of the inhibition zone was screened over the area represented by the yellow rectangle.B. Microscope picture of the analysed area (4.59 × 22.38 mm^2^).C. Average MALDI mass spectrum, recorded on the inhibition zone (4350 pixels, pixel step size 150 μm) over the mass range 990–1800 *m/z*. The inset represents a zoom on the mass range 990–1115 *m/z*, showing the signals of surfactins and iturins, detected as sodium [M+Na]^+^ and potassium [M+K]^+^ adducts. Red stars indicate sodium (*m/z* 1044.63) and potassium (*m/z* 1060.68) adducts of C_14_-surfactin, distribution of which is shown in D. Green stars indicate sodium (*m/z* 1058.63) and potassium (*m/z* 1074.67) adducts of C_15_-surfactin, distribution of which is shown in E. Yellow stars indicate sodium (*m/z* 1065.53) and potassium (*m/z* 1081.57) adducts of C_14_-iturin A, distribution of which is shown in F. Blue stars indicate sodium (*m/z* 1079.52) and potassium (*m/z* 1095.56) adducts of C_15_-iturin A, distribution of which is shown in G. Purple stars indicate sodium (*m/z* 1093.51) and potassium (*m/z* 1109.55) adducts of C_16_-iturin A, distribution of which is shown in H. Orange stars indicate sodium (*m/z* 1485.76) and potassium (*m/z* 1501.80) adducts of C_14_-fengycin B, distribution of which is shown in I. Scale Bar: 1 mm.Intensity scale is between 5% and 100%.

In order to get further insights into the nature of compounds involved in antifungal activity under these conditions, we exploited a MALDI-MS imaging assay recently developed (Debois *et al*., 2013). This technique allows very sensitive detection and spatial mapping of biomolecules coming from various origins (proteins, peptides, lipids, drugs and metabolites) at the surface of biological samples. Analysis of antibiotics accumulating in the growth inhibition zone, as illustrated in Fig. 6A, was performed after drying the semi-solid agar-based medium corresponding to the zone indicated in a vacuum desiccator and coating with the MALDI matrix (α-Cyano-4-hydroxycinnamic acid (CHCA)). Multiple individual molecular species were detected in this inhibition zone, but all corresponded to LPs as shown in the average MALDI mass spectrum recorded on a surface of 102 mm^2^ (Fig. 6C). Surfactin is detected in highest amounts, compared with iturin and fengycin. It is also noteworthy that surfactin does not exhibit the same localization as the two other LP families. Moreover, even C_14_ and C_15_ homologues of surfactin are not distributed the same way. C_14_-surfactin diffused from the bacterial colony into the medium, but stopped just before the front of migration of the fungus (Fig. 6D). On the other hand, C_15_-surfactin remained localized at distance from fungal mycelium and is more concentrated around the bacterial colony (Fig. 6E). These localizations demonstrate that surfactin is probably not involved in the antagonism against *Fusarium*, assuming that a strong inhibitory activity results in higher concentration close to the pathogen (on the right of the picture, Fig. 6A). This discrepancy between C_14_ and C_15_ localizations may be explained by different diffusion rates. Although secreted at the same time, the C_14_ homologue, exhibiting a more amphiphilic trait than C_15_, is probably more ‘capable’ to diffuse into the medium. During the same period of time, C_14_ diffuses further in the surrounding environment than the C_15_. In the case of iturins, all homologues readily diffused to accumulate just before and at the front of mycelium arrest (Fig. 6F–H). These results demonstrate that iturin is mainly involved in the antagonism, which is also supported by the poor detection of fengycins (Fig. 6C). The low contrast observed for the image of fengycin (Fig. 6I) strongly suggests that this LP family does not accumulate in inhibitory concentration. Taken together, the MALDI-MS imaging results confirm that iturins are responsible for most of the inhibition of *Fusarium* growth and demonstrate that the production of fungitoxic LPs by *Bacillus* grown in a nutritional context closer to natural conditions than optimal in vitro media is possible. Our previous works showed that *in planta* secretion of iturin and fengycin by root colonizing cells does occur even if observed concentrations are much lower than for surfactin (Nihorimbere *et al*., 2012). The relative proportions observed *in planta* actually are reflected by those observed here via Mass Spectrometry Imaging (MSI) by growing *Bacillus* on tomato root exudates. *Bacillus* can grow on exudates to produce significant quantities of iturins, but whether these amounts are sufficient to inhibit the growth of soil-borne pathogens in time and place remains to be determined. Indeed, LP production can occur with different efficacy from one rhizosphere microsite to the other causing spatial heterogeneity in the inhibition of the pathogen. However, MSI data show that LPs, once secreted, readily diffuse into the surrounding medium rather than adhering to the producing cells. Further work is needed, but it is still clear that antagonism developed in natural conditions will depend on the potential of the strain considered to produce the various LPs by feeding on rhizodeposits in the peculiar physico-chemical conditions of the rhizosphere environment. From a technical viewpoint, this experiment further illustrate the potential of MALDI-TOF MS imaging as powerful method to identify antibiotics produced in limited amounts by rhizobacteria growing on plant root exudates. It allows determining which molecular species is involved in an antagonism, with another microorganism, avoiding time-consuming steps of extraction, purification and activity tests, which are still commonly used in microbiology. Imaging MS may therefore nicely complement other molecular approaches to better understand how *Bacillus* strains may act in the rhizosphere where the tritrophic interaction with the host plant and the pathogen takes place.

### Comparison with a *Paenibacillus* strain

**Figure 7:**
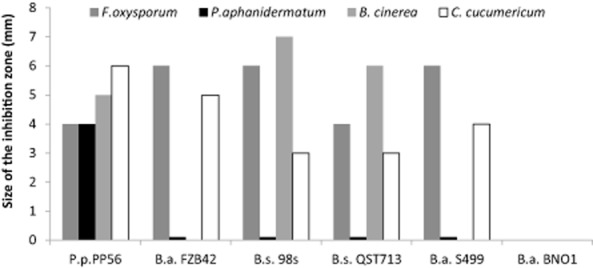
Intensity of the inhibition observed between *Paenibacillus polymyxa* Pp56 and several fungal pathogens compared with *Bacillus* strains. A second experiment displayed similar results.

**Figure 8:**
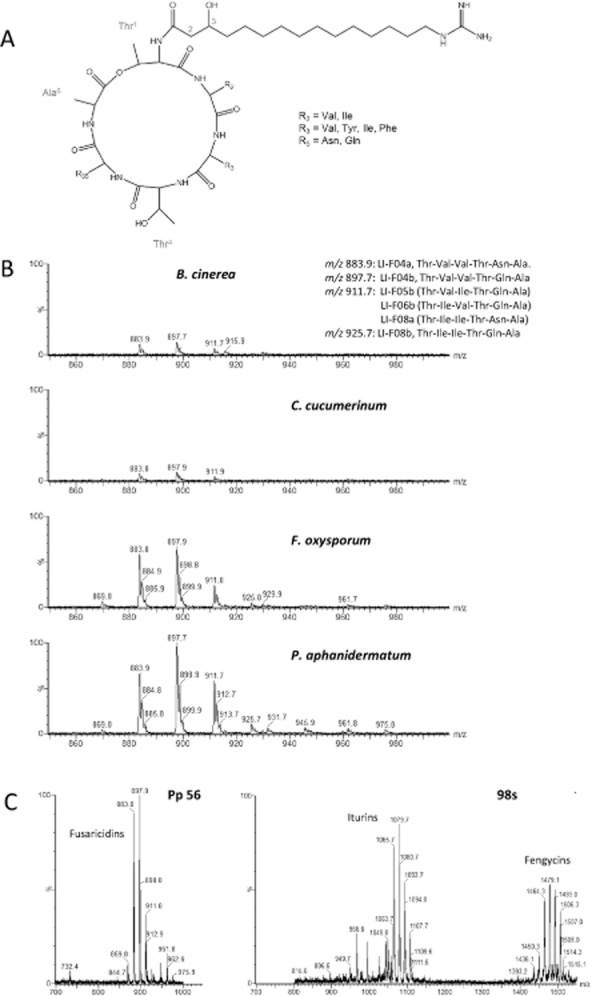
Antagonistic properties of *Paenibacillus polymyxa* correlates with accumulation of fusaricidins in the fungal inhibition zone.A. Common structure of fusaricidin-type lipopeptides also designated as LI-F compounds.B. Peaks observed in UPLC-ESI-MS for metabolite produced on gelified medium by *P. polymyxa* Pp56 antagonizing several fungal pathogens.C. Comparison of peak intensity between the fusaricidin-producing isolate PP56 and iturin and fengycin secreting *B. amyloliquefaciens* 98S. y Scales representing relative abundance of ions are linked.

Besides *Bacillus*, some isolates of the closely related *Paenibacillus* genus also display efficient biocontrol activity (McSpadden Gardener, 2004) and produce a vast array of structurally diverse antimicrobial compounds (Stein, 2005; Chen *et al*., 2009; Niu *et al*., 2011; Rückert *et al*., 2011). We therefore wanted to compare the behaviour of an isolate of *Paenibacillus polymyxa*, Pp56, with the observations on bacilli. Confrontation tests revealed that Pp56 displays a stronger and broader spectrum of antagonistic activity against fungal pathogens in comparison with the most efficient *Bacillus* isolates (Fig. 7). For instance, Pp56 is the sole isolate to significantly inhibit the growth of *P. aphanidermatum*, which was not affected by bacilli. Extraction and Ultra Performance Liquid Chromatography (UPLC)-Electrospray Ionization (ESI)-MS analysis of bacterial metabolites accumulating in the inhibition zone revealed several peaks corresponding to compounds in the mass range of LPs in amounts that are comparable with iturins and fengycins secreted by the best *B. amyloliquefaciens* producers. Based on their m/z ratio, these peaks corresponded to fusaricidin-type LPs also designated as LI-F compounds (Fig. 8) (Martin *et al*., 2003; Li and Jensen, 2008). Cellulase activity of Pp56 is similar to the tested bacilli (Fig. S4) and is therefore not responsible for the stronger antagonism exhibited by the strain towards *Pythium*. However, considering the broad-spectrum activity of fusaricidins (Kurusu *et al*., 1987; Lee *et al*., 2013), their production could be a good explanation for the strong and broad antagonistic potential observed for *P. polymyxa* Pp56 in the present work. A complementary work studied in detail the antagonism of Pp56 against *F. oxysporum* on natural root exudates (Debois *et al*., 2013). Results of this MS imaging study also strongly supported a major role for fusaricidin-type LPs. First, spatio-temporal mapping of antibiotics accumulating in the inhibition zone between strain Pp56 and *Fusarium* revealed a clear limit for accumulation of fusaricidin B and LI-F05b/ 06b/ 08a that corresponded exactly to the area of inhibition of fungal mycelium. Second, other antibiotics such as lantibiotics (He *et al*., 2007; Huang and Yousef, 2012), macrolides (Wu *et al*., 2010) and polyketides (Niu *et al*., 2011) were not detected at any time-point in the growth inhibition zone examined either by MALDI imaging or by UPLC-MS after extraction.

## Experimental procedures

### Bacterial strains

*Bacillus* strains S499 (Ongena *et al*., 2005a,b) and GA1 (Arguelles-Arias *et al*., 2009) have been previously described. Strain FZB42 and its mutants were kindly provided by R. Borriss of Humboldt University, Berlin. Isolates QST713/QST2808 were graciously provided by J. Margolis of the Agraquest society. The BNO1 and ATCC strains 6633 and 21332 were obtained from the laboratory collection. *Bacillus* strains 98S, 23, 67, 164, 98r, 104, III and *Paenibacillus* strain 56 were kindly provided by Professor B. McSpadden Gardener from Ohio State University.

### Agitated cultures

Bacteria were streaked on Luria medium (per litre: 8 g of peptone; 4 g of yeast extract; 4 g of NaCl; 0.8 g of glucose; 12.5 g of agar) and incubated overnight at 30°C. These colonies were used to inoculate Erlenmeyer flasks (250 ml) containing 50 ml of a medium optimized for LP production, Opt medium (Jacques *et al*., 1999). These flasks were shaken during 3 days at 30°C.

### ULPC-MS analysis

Samples were analysed by reverse phase UPLC–MS (UPLC, Waters, Acquity class H) coupled with a single quadrupole MS (SQDetector, Waters, Acquity) on an Acquity UPLC BEH C18 (Waters) 2.1 × 50 mm, 1.7 μm column. We used a method, based on acetonitrile gradients, that allowed the simultaneous detection of all three LP families. Elution was started at 30% acetonitrile (flow rate of 0.60 ml min^−1^). After 2.43 min, the percentage of acetonitrile was brought up to 95% and held until 5.2 min. Then, the column was stabilized at an acetonitrile percentage of 30% for 1.7 min. Compounds were identified on the basis of their retention times compared with authentic standards (98% purity, Lipofabrik society, Villeneuve d'Asc, France) and the masses detected in the SQDetector. Ionization and source conditions were set as follows: source temperature, 130°C; desolvation temperature, 400°C; nitrogen flow, 1000 l h^−1^; cone voltage, 120 V.

### Antagonism assays

The pathogens used in this work were *Cladosporium cucumerinum*, *B. cinerea*, *F. oxysporum* and *Pythium aphanidermatum*. These fungi were maintained at 25°C by sub-culturing every 2 weeks on PDA medium (Potato dextrose agar). Cultures that were 15 days old were used to inoculate PDA test plates. *Fusarium oxysporum* and *P. aphanidermatum* were inoculated as central plugs on test plates. *Cladosporium cucumerinum* and *B. cinerea* were scrapped using salt peptone water (per litre: 5 g of NaCl; 1 g if peptone; 2 ml of Tween 80) that was then filtered to eliminate mycelium debris. A volume of 100 μl of the obtained suspension (10^6^ spore ml^−1^) was platted on the test medium. Active bacterial populations were obtained by overnight culture on solid Luria medium at 30°C. Some of the obtained cells were picked up with a sterile toothpick and deposited at 2 cm from the border of the test plate. Test media were incubated overnight at 30°C. The incubation was continued at 25°C during 2 days. Antagonism was quantified as radius of the inhibition zone, thus the distance (in mm) between the fungus and the bacterial colony.

### LP quantification in the inhibition zone

Agar samples were taken from the inhibition zone. Weight samples, around 150 mg of agar, were mixed with 500 μl of acetonitrile : water (1:1; v : v). This mix was sonicated (BandelinSonoplus HD 2070) twice during 30 s at 30% of the power of the device. Next, samples were homogenized (vortex) and then centrifuged and filtered to eliminate any particles. Obtained filtrates were analysed using UPLC-MS (see above).

### LP production and antagonism on natural root exudates

The experiment was conducted as described by Debois and colleagues (2013). Briefly, the fungus *F. oxysporum* was confronted with the *Bacillus* strain on agar-based medium containing tomato root exudates (obtained through hydroponic culture). The *Bacillus* was streaked, as one line, at the centre of the plate. Two days later, the fungus was deposited as two plugs on the borders of the plate and two plugs near the bacterial line. After 3 days, the plates were dried under vacuum and coated with the MALDI matrix. The 5 mg ml^−1^ CHCA solution in Acetonitrile (ACN)/0.2% Trifluoroacetic acid (TFA) 70:30 was sprayed onto the agar plate using an automated sprayer (ImagePrep, Bruker Daltonics, Germany). For MALDI-MS imaging experiments, a MALDI-TOF/TOF (UltraFlex II, Bruker Daltonics, Germany) instrument was used to record mass spectra resulting from the accumulation of 300 laser shots. The pixel size was set to 150 μm. Before launching the acquisition, 1 μl of a LPs mixture was deposited next to the sample and was used to internally calibrate the instrument (error inferior to 4 p.p.m.). After the acquisition, the whole data set was submitted to a processing method including smoothing, baseline correction and mass re-calibration. Ion images were generated from normalized data using FlexImaging 2.1 (Bruker Daltonics, Germany) with a mass filter width fixed at 0.1 Da.

In this work, before MALDI analysis, the antagonism intensity was assessed as follows. The sizes of all fungal spots were measured in millimetres. The fungal spots closest and most distant from the bacterial line were considered respectively as inhibited spots and reference spots. Antagonism intensity was calculated as the following ratio, Sr being the mean of the sizes of the reference spots and Si the same mean for the inhibited spot: (Sr − Si)/Sr. In a separate experiment, the same assay was conducted on root exudates from zucchini and bean plants obtained as for tomato exudates.

### Cellulase activity test

Enzymatic activities were assessed in a qualitative way through halo formation on solid media. To obtain active cell populations, all strains were streaked on solid Luria medium and incubated overnight at 30°C. The obtained bacterial populations were used to directly inoculate the enzyme test medium. The cellulase test medium is composed of Plate Count Agar (PCA) medium (5 g of peptone, 2.5 g of yeast extract, 1 g of glucose and 15 g of agar in 1 litre distilled water) complemented with 0.1% azurine cross-linked (AZCL)-cellulose. Incubation was conducted at 30°C. The presence of halos was checked after 48 h. The AZCL-cellulose used in the medium is insoluble; the enzymatic digestion of the cellulose releases the AZCL dye generating bleu halos. Results were expressed as the ratio of the diameter of the halo by the diameter of the colony.

## Conclusions

Globally, our data illustrate the benefit for the antagonistic potential of bacilli to form both iturins and fengycins. Although surfactin is poorly active directly on fungal aggressors, secretion of this LP favours root tissue colonization and rhizosphere establishment of the bacterium, which is a prerequisite for consistent release of antifungals and successful biocontrol of phytopathogens. In addition, surfactin represents one of the rare *Bacillus* products identified so far, as elicitor of plant immunity. A major role for surfactin in the ISR-triggering efficacy of *Bacillus* strains has indeed been demonstrated on bean and solanaceae in our previous works (Ongena *et al*., 2007; Jourdan *et al*., 2009; Pertot *et al*., 2013; Cawoy *et al*., 2014). Efficient co-production of all three families is thus clearly advantageous and it is not surprising that this trait occurs in the most efficient *B. amyloliquefaciens* isolates brought to the market as biocontrol agents. The LP signature in terms of diversity and relative production could thus be used as first screening criterium for the selection of potential new biocontrol strains and to predict antagonistic potential of bacilli in different pathosystems. In an evolutionary perspective, it may also explain the high if not invariable occurrence of the three LP operons in natural isolates of this *B*. *amyloliquefaciens* species (Hamdache *et al*., 2011; Mora *et al*., 2011) that must establish and maintain in the highly competitive rhizosphere ecological niche.

## Supporting Information

**Fig. S1.** LC-ESI-MS profiling of lipopeptides secreted by the isolates used in this work in the inhibiton zone formed against *Fusarium oxysporum* on PDA (see Methods section). Similar profiles were produced by the bacilli during confrontation with the other fungi. For each lipopeptide family, several peaks are detected which correspond to the various co-produced homologues differing in the length/isomery of the fatty acid tail. For each strain, Y axes in the mass spectra for surfactins, iturins and fengycins represent total ion current values (relative abundance) and were linked at the same scale to allow comparison of the relative intensities of the ions correponding to the three LP families based on peak area/height. Data were obtained from analysis of extracts prepared from one culture but similar MS spectra and LP profiles were obtained in a biological repeat. **Fig. S2.** Correlations between LP concentration in the inhibition zone and intensity of the antagonism. Iturin concentration against antagonism intensity for: A *Fusarium oxysporum*, B *Cladosporium cucumerinum*, C *Botrytis cinerea* and D *Pythium aphanidermatum*. Fengycin concentration against antagonism intensity for the same pathogens respectively in panels E, F, G and H. Panels I and J show the correlation of antagonism intensity against *Botrytis cinerea*related to the combined concentration of iturin and fengycin. These last two panels show different concentration ranges. Each panel also includes an illustration of the observed antagonisms for the concerned fungus. Agar samples were taken from the inhibition zone and analysed for LPs content. Antagonism intensity was evaluated in direct confrontation tests between Bacilli and phytopathogens on PDA medium and quantified by measuring the radius of the inhibition zone between the PGPR and the fungi. Presented data are means from two biological repeats. **Fig. S3.** Lipopeptides may interact to form a precipitate. A. Illustration of the «white line» observed around some of the colonies co-producing all three LP families during antagonism against *Botrytis cinerea*. The development of such «white line» upon confrontation with *Botrytis* was observed for strain FZB42 but also for all strains co-producing the three LP families but not for co-producers of surfactin and fengycin (natural strains) or for the fengycin and iturin coproducing mutant CH1. Circles (a, b and c) indicate the three sampling zones used for each strain. B. Chromatograms illustrating the differential LP concentrations observed in the three sampling zones. Agar samples were collected in the area corresponding to the line or out of this zone and UPLC-MS revealed a much higher LP content in the first sample. More specifically, it corresponded to a major enrichment in surfactin and fengycin (4 to 13 times concentrated) and a lower concentration factor for iturin (2 times). Shown data are representative of two biological repeats. Similar tendencies in LP distribution were observed for all strains displaying a ‘white line’. **Fig. S4.** A. Cellulase production potential of the seven strains. The cellulase test medium is composed of a rich culture medium supplemented with AZCL-cellulose. This molecule is insoluble; the enzymatic digestion of the cellulose releases the AZCL dye generating blue halos. Results were expressed as the ratio of the diameter of the halo by the diameter of the colony. The presented data are mean and standard deviation calculated from two biological repeats. B. Illustration of the formation of bleu halos on the cellulase test medium.
