# Supplementary material for: Lipopeptides as main ingredients for inhibition of fungal phytopathogens by Bacillus subtilis/amyloliquefaciens
Source: Microb Biotechnol. 2014 Dec 19;8(2):281–95. doi: 10.1111/1751-7915.12238 (PMC4353342; doi:10.1111/1751-7915.12238)

**Supporting information**

**Figure S1.** LC-ESI-MS profiling of lipopeptides secreted by the isolates used in this work in the inhibiton zone formed against *Fusarium oxysporum* on PDA (see Methods section). Similar profiles were produced by the bacilli during confrontation with the other fungi. For each lipopeptide family, several peaks are detected which correspond to the various co-produced homologues differing in the length/isomery of the fatty acid tail. For each strain, Y axes in the mass spectra for surfactins, iturins and fengycins represent total ion current values (relative abundance) and were linked at the same scale to allow comparison of the relative intensities of the ions correponding to the three LP families based on peak area/height. Data were obtained from analysis of extracts prepared from one culture but similar MS spectra and LP profiles were obtained in a biological repeat.

**
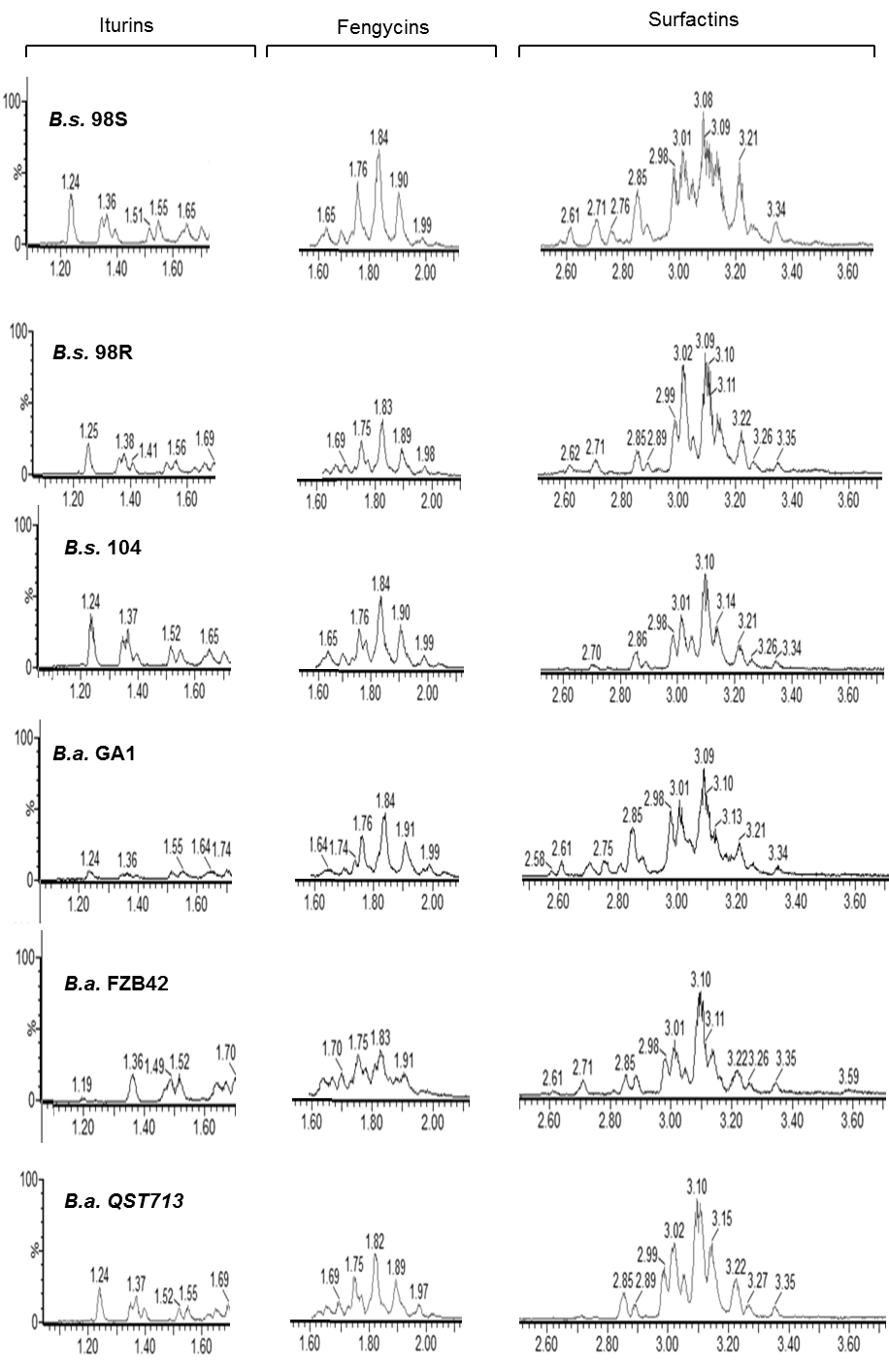
**

**
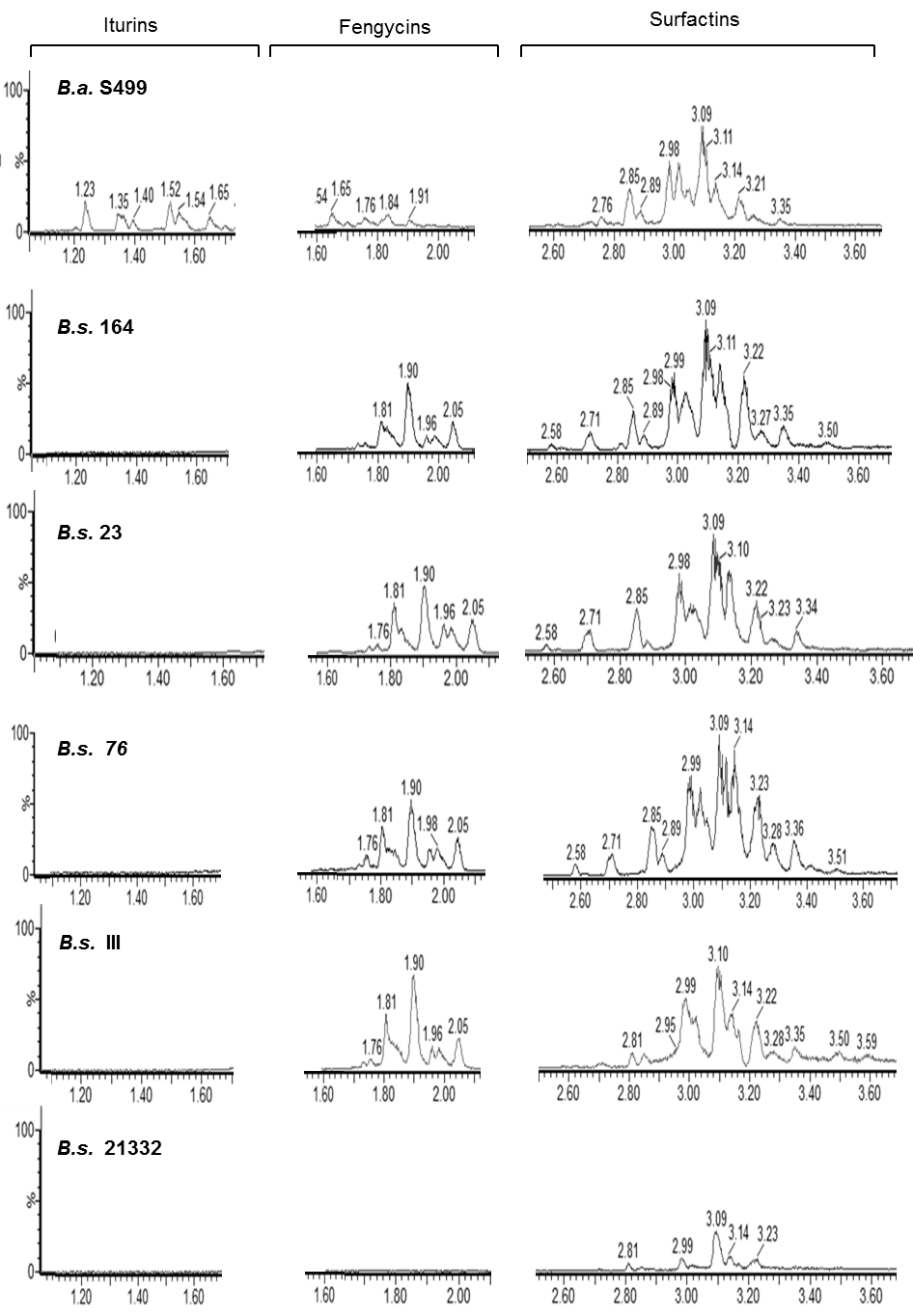
**

**
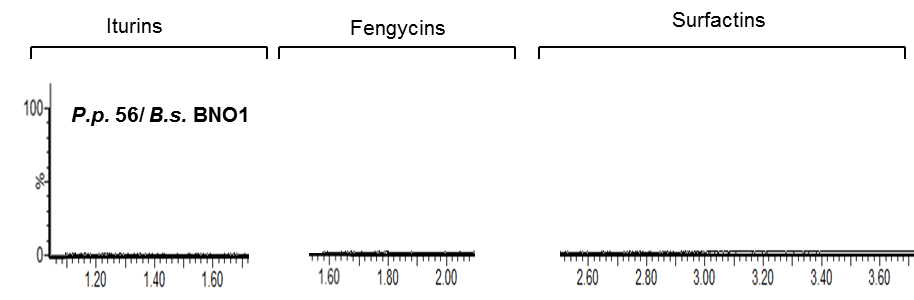
**

**Figure S2.** Correlations between LP concentration in the inhibition zone and intensity of the antagonism. Iturin concentration against antagonism intensity for: **A** *Fusarium oxysporum,* **B** *Cladosporium cucumerinum*, **C** *Botrytis cinerea* and **D** *Pythium aphanidermatum*. Fengycin concentration against antagonism intensity for the same pathogens respectively in panels **E, F, G** and **H**. Panels **I** and **J** show the correlation of antagonism intensity against *Botrytis cinerea*related to the combined concentration of iturin and fengycin. These last two panels show different concentration ranges. Each panel also includes an illustration of the observed antagonisms for the concerned fungus. Agar samples were taken from the inhibition zone and analyzed for LPs content. Antagonism intensity was evaluated in direct confrontation tests between Bacilli and phytopathogens on PDA medium and quantified by measuring the radius of the inhibition zone between the PGPR and the fungi. Presented data are means from two biological repeats.

**
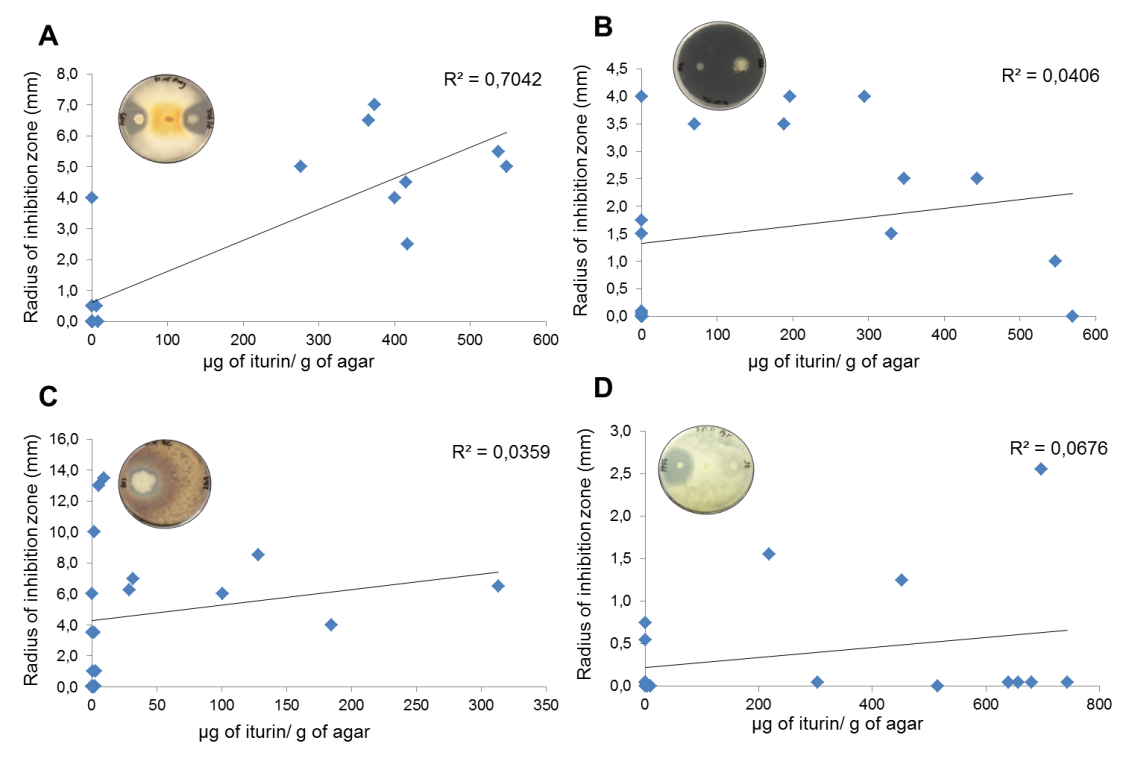
**

**
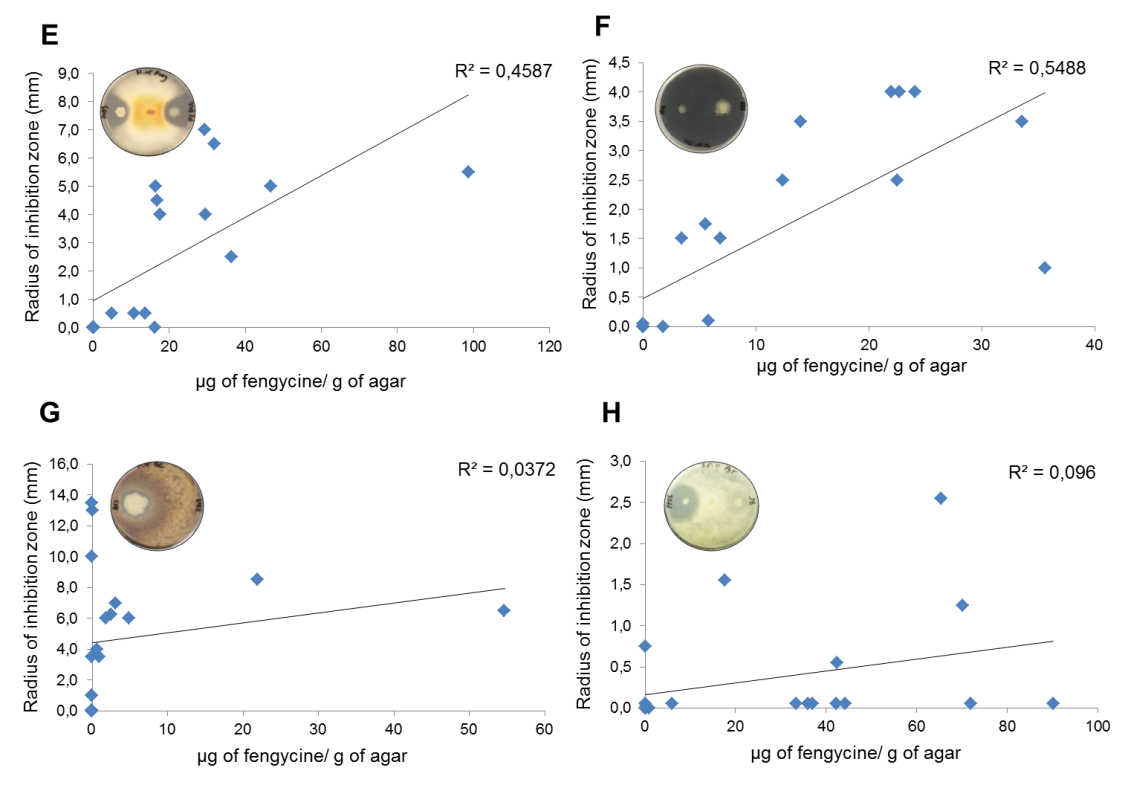
**

**
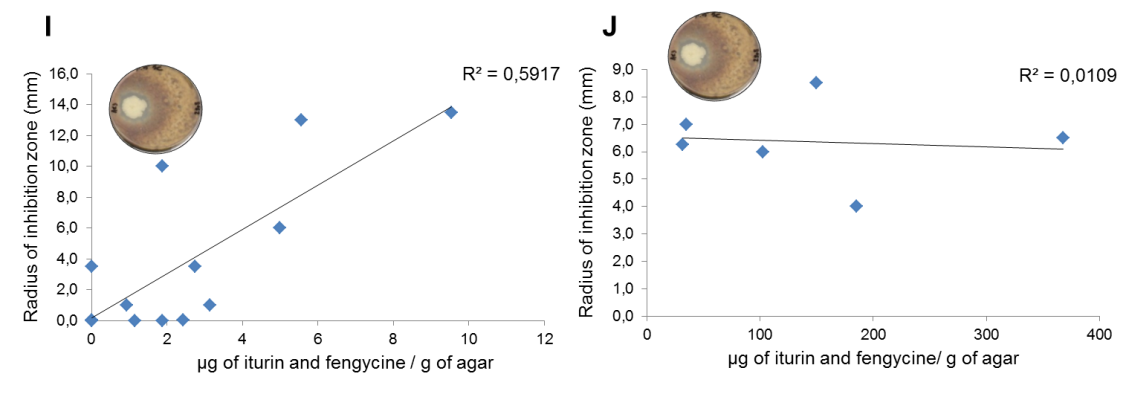
**

**Figure S3.** Lipopeptides may interact to form a precipitate. **A.** Illustration of the «white line» observed around some of the colonies co-producing all three LP families during antagonism against *Botrytis cinerea*. The development of such «white line» upon confrontation with *Botrytis* was observed for strain FZB42 but also for all strains co-producing the three LP families but not for co-producers of surfactin and fengycin (natural strains) or for the fengycin and iturin coproducing mutant CH1. Circles (a, b and c) indicate the three sampling zones used for each strain. **B.** Chromatograms illustrating the differential LP concentrations observed in the three sampling zones. Agar samples were collected in the area corresponding to the line or out of this zone and UPLC-MS revealed a much higher LP content in the first sample. More specifically, it corresponded to a major enrichment in surfactin and fengycin (4 to 13 times concentrated) and a lower concentration factor for iturin (2 times). Shown data are representative of two biological repeats. Similar tendencies in LP distribution were observed for all strains displaying a “white line”.


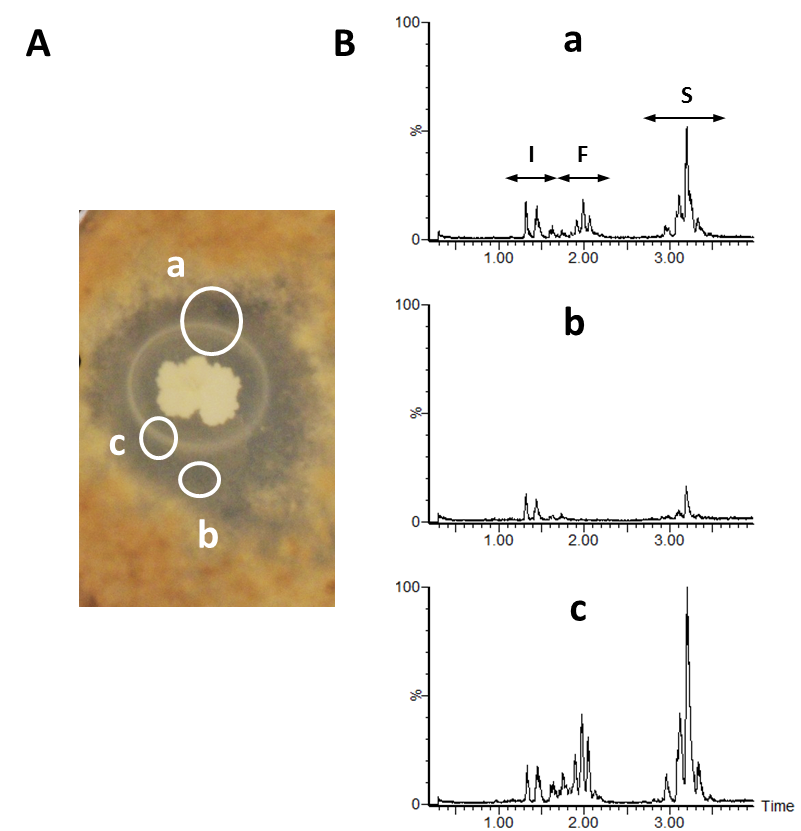


**Figure S4. A.** Cellulase production potential of the seven strains. The cellulase test medium is composed of a rich culture medium supplemented with AZCL-cellulose. This molecule is insoluble; the enzymatic digestion of the cellulose releases the AZCL dye generating blue halos. Results were expressed as the ratio of the diameter of the halo by the diameter of the colony. The presented data are mean and standard deviation calculated from two biological repeats. **B.** Illustration of the formation of bleu halos on the cellulase test medium.


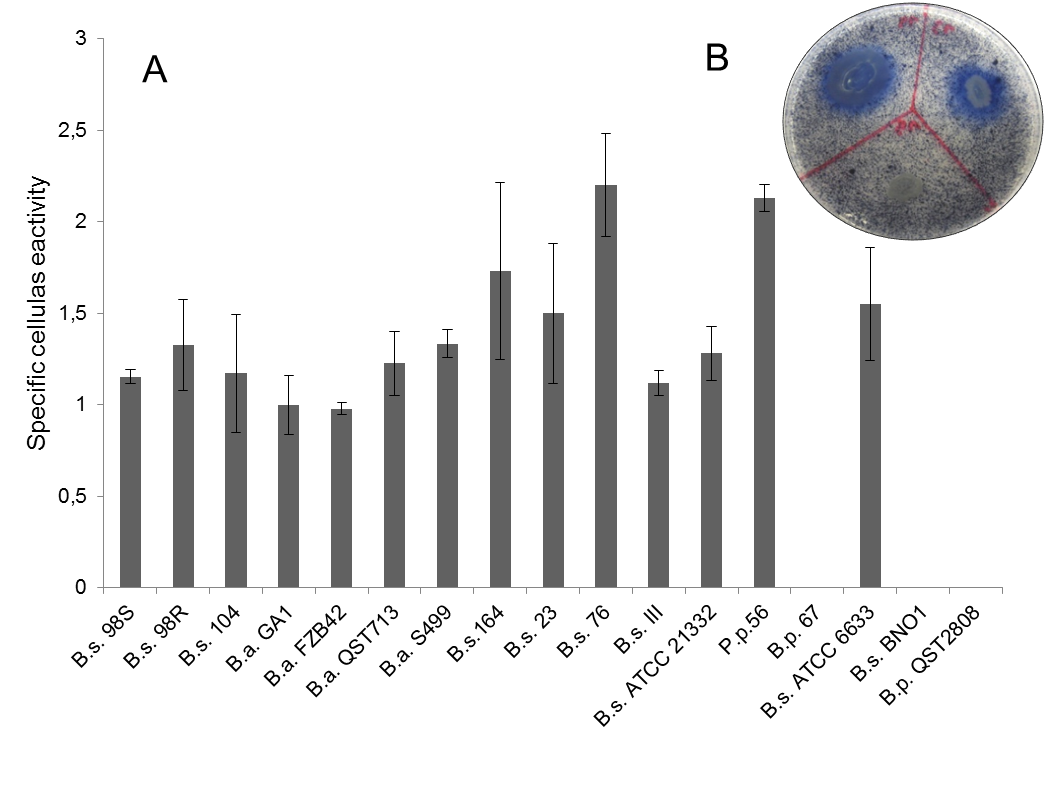

Supplement: Supplementary file 1 [file mbt20008-0281-sd1.docx]
